# Organoids in Lung Cancer Management

**DOI:** 10.3389/fsurg.2021.753801

**Published:** 2021-12-09

**Authors:** Yushi Li, Joyce W. Y. Chan, Rainbow W. H. Lau, Winnie W. Y. Cheung, Alissa Michelle Wong, Aikha M. Wong, Nathalie Wong, Calvin Sze Hang Ng

**Affiliations:** Department of Surgery, Prince of Wales Hospital, The Chinese University of Hong Kong, Shatin, Hong Kong SAR, China

**Keywords:** lung cancer, organoids, drug screening, disease model, preclinical model

## Abstract

Lung cancer is a complex milieu of genomically altered cancer cells, a diverse collection of differentiated cells and nonneoplastic stroma. Lung cancer organoids is a three-dimensional structure grown from patient cancer tissue that could mimic *in vivo* complex behavior and cellular architecture of the cancer. Furthermore, the genomic alterations of the primary lung tumor is captured *ex vivo*. Lung cancer organoids have become an important preclinical model for oncology studies in recent years. It could be used to model the development of lung cancer, investigate the process of tumorigenesis, and also study the signaling pathways. The organoids could also be a platform to perform drug screening and biomarker validation of lung cancer, providing a promising prediction of patient-specific drug response. In this review, we described how lung cancer organoids have opened new avenues for translating basic cancer research into clinical therapy and discussed the latest and future developments in organoid technology, which could be further applied in lung cancer organoids research.

## Background

Lung cancer remains the leading cause of malignancy-related mortality world-wide (1), and is one of the most prevalent cancer in the world (2). It could be histologically divided into three major types: nonsmall cell lung cancer (NSCLC), small cell lung cancer (SCLC), and others. Among these three types, NSCLC is the dominant type of lung cancer (3). In general, the treatment outcomes and prognosis of patients with NSCLC is poor (4). There is lack of a valid model which could accurately represent the heterogeneity of the original lung tumor and the treatment response (5). So far, several models for lung cancer treatment research are available, such as two-dimensional (2D) culture lung cancer cell lines, xenograft models *in vivo*, patient-derived xenograft (PDX) models, and also three-dimensional (3D) culture organoids models. Large-scale genomic analyses of lung cancer have shown substantial phenotypic and genetic heterogeneity between individuals represented by intertumoral and intratumoural heterogeneity (6), which makes it difficult to study genetic and molecular alteration in lung cancer *via* lung cancer cell lines. After the long-term culture and propagation, cancer cell lines often display genetic artifacts from *in vitro* passaging. This genetic drift is also a big limitation for patient-oriented drug screening and clinically relevant explorations *via* lung cancer cell lines. Besides, model in animals and PDX also has its limitations, such as engraftment efficiencies, the limited passages in mice, species differences, and the expensive cost to establish as a model. Patient-derived cancer organoids models have become a significant preclinical model for several cancer studies like colon cancer, breast cancer, prostate cancer, pancreas cancer, and lung cancer. In this work we are going to describe the advantages and disadvantages of this advanced cancer study model as well as its latest technology.

## Comparison Between Different Types of Lung Cancer Study Models

There are several kinds of preclinical models for lung cancer research and study. For example, lung cancer cell lines models, PDX, and lung cancer organoids models. Each type of models has strengths and shortcomings, respectively, when used to study and characterize the genotype and phenotype of lung cancer. How to choose the models for studies should depend on the experimental goal (Table 1).

**Table 1:** Comparison between different types of lung cancer study models.

	**Lung cancer cell lines**	**PDX model**	**Organoids model**
Advantage	Easy handling; low cost; rapid culturing; large-scale usage.	Well retain genomic and phenotype of original patient specimens.	Well maintain the biology and genetic characteristics of original cancer; well mimic the microenvironment of initial tumor; less time consuming than PDX model; could be genetically modify.
Disadvantage.	Heterogeneous between different lab; genetic drift and clonal selection after long-term layer culture; lost the key characteristic of original tumor.	Expensive and time-consuming; limited passage number; lack of human TME cells.	Lack of standardized methodology; lack of vasculature component; more difficult of long-term initial and propagation.

### Lung Cancer Cell Lines Models and Its Clinical Applications

The advantages of lung cancer cell lines model include their ease of handling, low cost, and rapid culturing, which make them popular in numerous lung cancer research.

Firstly, lung cancer cell lines were used to explore the drug sensitivity and drug resistance in multiple studies. For instance, some lung cancer cell lines, such as H3255(EGFR L858R) and PC9(EGFR del19), demonstrated sensitivity to epidermal growth factor receptor tyrosine kinase inhibitors (EGFR TKIs) Osimertinib, rociletinib, erlotinib, and afatinib, whereas HCC827(EGFR del19) is sensitive to gefitinib or erlotinib or the EGFR inhibitory antibody cetuximab. Previous works showed that the proliferation of cell lines with anaplastic lymphoma kinase (ALK) fusion, such as cell line H3122 (harboring ALK-EML4), could be suppressed by ALK-inhibitors like TAE684 (7). However, there are some disadvantages of lung cancer cell lines models. They do not always keep the characteristics of original lung cancer. Due to the clonal drift and expansion and the monolayer growth behavior on a plastic surface, some immortalization genetic changes can occur, which might change the phenotype like drug sensitivity of lung cancer cell lines. For example, some studies showed that NCI-H1975 bears T790M mutation, which may confer resistance to afatinib. But for NCI-H1975 which carries L858R mutation, it shows sensitivity to new-generation TKIs like rociletinib, osimeritnib, and afatinib. Cellosaurus cell line DFCI032 showed drug resistance to TAE684 due to the coactivation of EGFR receptor and Erb-B2 receptor tyrosine kinase 2 (ERBB2). But its proliferation could be suppressed by the combination of TEA684 and EGFR/ERBB2 kinase inhibitor CL-387,785 (7). Besides, because of the low cost and ease of culturing, large-scale studies like cancer cell line encyclopedia (8), the genomics of drug sensitivity in cancer (9–11), the cancer therapeutics response portal (12), the connectivity map (13, 14), and the genentech cell line screening initiative (15, 16) were performed in lung cancer cell lines to detect their genomic, copy number variant, transcriptomic, and drug response. At the same time, these large-scale studies identified the associations between molecular maker and drug sensitive as well as the gene expression level after treatment.

Secondly, lung cancer cell lines are relatively easy to handle when used to construct certain genetic operations, for example, lenti-virus and CRISPR Cas9 systems, compared with using PDX and organoids model. In addition, the rapid growth of cancer cell lines saves time and labor, and could screen out stable clones and cell subtypes. Numerous studies have used CRISPR Cas9 technique to knock out a specific gene in lung cell line to explore the synthetic lethality and driver mutation. For example, a study has used CRIPSR Cas9 to knock out the EGFR-mutation in H1975 cell lines and the wild-type EGFR alleles in A549 cell line. The study showed that the proliferation ability and tumor volumes of xenografts was suppressed only in NCI-H1975 cell line, which provided a treatment strategy to disrupt oncogenic mutation during cancer therapy (17). Nevertheless, the disadvantage of lung cancer cell line for exploring therapeutic strategies is obvious: lung cancer cell lines can be very different from the original cancer in genetic or epigenetic form due to the lack of differentiation during long-term culturing, survival pressure selecting, monolayer culturing, and culturing without interacting with original microenvironment cells, like stromal cell, immune cell, and also inflammatory cells (18). Previously, research has shown that the expression level of surface markers like TTF1 and TP63 was similar in lung adenocarcinoma cell lines and lung squamous cell lines, which was significantly different from clinical histopathology results which show that lung squamous was negative for TTF1 and approximately 100% positive for TP63, whereas lung adenocarcinoma was 70–80% positive for TTF1 (8). Furthermore, the same cell lines from different laboratories are not totally identical due to the different experiment conditions and poor consistency between different large-scale lung cancer cell lines and drug sensitivity, which have been reported (19). The absence of intercellular interaction in lung cancer cell line cultures also limits its potential use in translational medicine study. Therefore, although there are numerous advantages for lung cancer cell lines model, its disadvantages still limit the translation between lung cancer cell lines model and clinical application.

### PDX Models

#### Advantages of Lung Cancer PDX Models

Patient-derived tumor xenograft models are developed by grafting and propagating patient-derived lung cancer tissues in immunodeficient or humanized animals, like mice. These patient-derived tissues growing in a three-dimensional *in vivo* microenvironment display vasculature that could provide oxygen and nutrients. The direct implantation of human cancerous tissue circumvents *in vitro* genetic drift and clonal selection. The *in vivo* microenvironment also allows cell–cell interaction and communication with host stromal cells and immune cells. These features enable PDX model to retain most of the genomic and phenotype of the derived patient specimen. Therefore, PDX is a great preclinical model for investigating drug response and a promising indicator for clinical therapies. It also is a significant laboratory tool to investigate the mechanisms of drug resistance.

Previous studies have shown that NSCLC PDX models with EGFR exon19 deletion mutation and L858R mutation was sensitive to EGFR TKIs gefitinib, erlotinib, dacomitinib, and afatinib, which was in accordance with the clinical results (20). In addition, PDX models with EGFR exon19 deletion and T790M mutations were sensitive to cetuximab but showed no response to gefitinib and erlotinib (21). Furthermore, PDX models were able to demonstrate that KRAS mutation showed drug resistance to gefitinib, which was consistent with clinical outcome (22). Multiple studies have also shown that PDX models with ALK rearrangement had drug sensitive to ALK inhibitor (23), and if PDX models with ALK rearrangement showed drug resistance toward ALK inhibitor, the patient would also display similar drug response to ALK inhibitor (24). More specifically, in lung squamous cancers, PDX models with PI3CA E542K mutation showed drug sensitivity to phosphoinositide 3-kinase (PI3K) inhibitor (25). Conventional chemotherapies also produced similar therapeutic response toward NSCLC and SCLC in clinical patients and in correlated PDX models (26, 27). Furthermore, the effects of synergy between chemotherapies and the enhancer of zeste homolog 2 (EZH2) inhibition on tumor-inhibition of SCLC was demonstrated in PDX models, even in those that were resistant to chemotherapy (28). PDX models with lung cancer have also been utilized to explore immunotherapy in lung cancer. For example, anti PD-1 checkpoint inhibitors led to the suppression of tumor growth in humanized mice engrafted with A549 cell lines, which provides an advanced platform for evaluation in immunotherapy (29).

#### Disadvantages of Lung Cancer PDX Models

Although there are plenty of advantages of PDX models for lung cancer research, there are some limitations too. For instance, large-scale study is more difficult to perform in PDX models when comparing with lung cancer cell lines models. Besides, construction of PDX models might need more time and are more expensive when compared with lung cancer cell lines models and organoids models. Furthermore, the limited passage number due to a probable adaptive genetic evolution to the murine background in PDX models remained a problem for lung cancer research (30). Last but not least, the replacement of human immune cells or stromal cells by mouse stromal cells in nonhumanized mice after several passage could lead to the lack of cell–cell interaction and communication between tumor cells and the original human stroma (31). These disadvantages of PDX models remain a challenge. To improve the methodology of PDX model construction would be a significant way to better simulate the patient tumor and microenvironment, and at the same time to lower the cost and time.

### Patient-Derived Lung Cancer Organoids Models

#### Introduction of Organoids Models in Lung Cancer

“Lung cancer organoids” refers to the 3D structures processed from lung tumor tissues, containing multiple cell types and growing in an organized manner (32). Lung cancer cell line derived from patient tumor tissues contain multiple lung cell types, not only the cancer cells in different stages but also the stromal cells. Besides, short-term cultured cells could capture the latest stage of tumor, which could represent the genetic characteristic of the original tumor (33). Although a part of the lung patient-derived cell line could grow in a monolayer, the original heterogeneity and 3D organ structure could not be preserved in the monolayer differentiation environments.

Normal function of human organ depends on the synergistic interaction of multiple cell types, which are distributed and organized within a 3D structure (33). Like normal organ, lung tumor is a complex community. The growth of the tumor is also supported by a complex extracellular matrix and the tumor microenvironment (TME). To overcome the limitations of monolayer cultures from patient-derived lung cancer cells, strategies have been recently developed with a miniature 3D structure model named “organoids.” By using the organoids, patient-derived lung cancer cell lines can be supported by an extracellular matrix, which could better mimic the complexity and architecture *in vivo*.

#### Lung Cancer Organoids Culturing Technology

**Figure 1:**
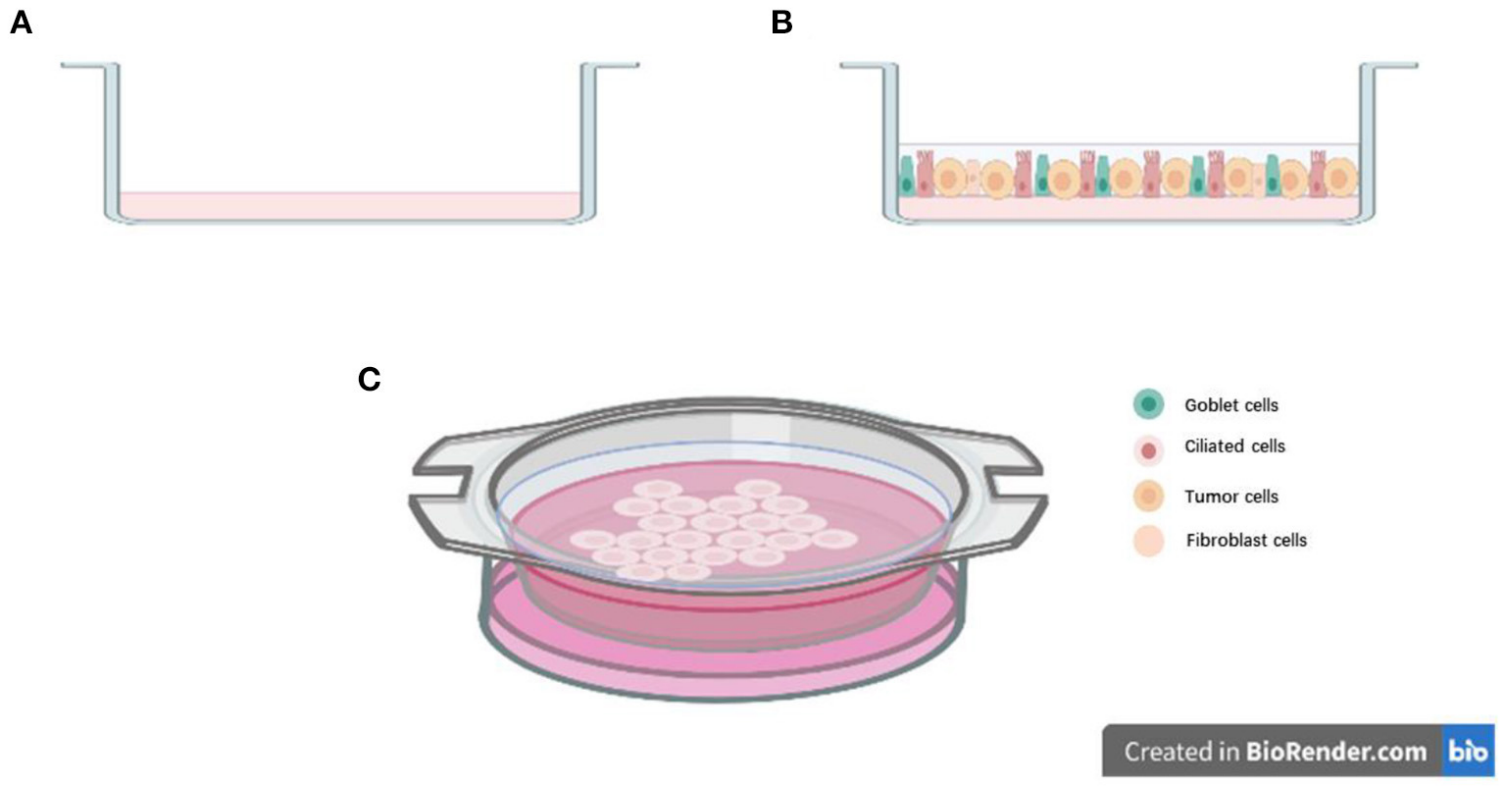
Air-liquid interface (ALI) culture system: **(A)** Precoat collagen gel in a 0.4 μm transwell; **(B)** Resuspend cells with collagen gel and layered on top of presolidified collagen gel; **(C)** Place the transwell into cell culture dish with culture medium.

To construct a 3D structure, a “scaffold” for growth is required, and organoids were mostly cultured in Matrigel (34). The other important consideration in organoid culture is the supplement of growth factors and small molecular inhibitors (like Y-27632) or activators (like SAG) used, which can vary from laboratory to laboratory. These small molecular inhibitors and activators could inhibit or activate different pathways, which could help organoids growing and maintaining their phenotype. For example, Tsao et al.'s organoid study utilized common culture medium formulations like ADMEM/F12, Glutamax, HEPES, and pen-strep, and also *N*-acetyl-L-cysteine (NALC), B-27 supplement to maintain the stem cell properties of patient-derived lung cancer cells. But in addition, they added small molecular inhibitors or activators into the culture medium, which included A83-01, Y-27632, Noggin, CHIR 99021, and Smoothened agonist (SAG). In their tailored “cocktail,” some growth factors including fibroblast growth factor 4 (FGF 4), FGF 10, and epidermal growth factor (EGF) were also added (35). However, in contrast, Jang et al.'s organoid study culture medium components were much simpler that included only B-27 supplement, Y-27632, bFGF, EGF, N2 supplement, and basic formulations like DMEM/F12 and pen-strep (36). Both their lung cancer organoids were seeded in a growth factor reduced Matrigel. Their successful culture periods were more than 3 months, and in Jang' s study, lung cancer organoids could be successfully cultured for more than half a year. The difference in experience from the two groups remind us that the addition of culture medium components is only partly responsible for organoid culture success. In fact, the problem of overgrowth of normal epithelial cells during lung cancer organoids cultures are frequently reported (37). Some studies showed that the growth advantage of normal lung cells during organoids culture may be because of the genetically unstable nature and the high apoptosis rate of cancer cells (38). Besides, the culture medium with much stem cell formulations could be another reason. For example, in Clever et al.'s study, although they added many small molecular signaling modulators and grow factors into the culture medium like R-spondin, Noggin, SB 202190, A83-01, Y-27632, FGF7, and FGF10, they also added Nutlin-3a into the medium to drive TP53 wild-type into senescence or apoptosis, which could supress the rapid growth of normal epithelium cells (39). By using Nutlin-3a selection, they have selectively expanded the lung cancer organoids with P53 mutation and have successfully established pure lung cancer organoids from different histological subtypes. But the culture time of their successful study was not reported. The organoids culture methods mentioned above are aiming at established pure lung cancer organoids or restrain the normal lung cells as much as possible. However, some research has performed an air–liquid interface (ALI) culture method for lung cancer organoids to conserve more lung stroma cells. For instance, in Kuo et al.'s study, the formulation of their medium is much similar with Clever et al.'s. They both include ADMEM/F12, NALC, B-27 supplement, R-spondin, Noggin, Nicotinamide, A 83-01, and SB-202190, but instead of Y-27632, FGF 7, and FGF 10, Kuo et al. also added EGF, Gastrin, and Wnt3a into the culture medium. More importantly, they used rat tail type 1 collagen to set up the ALI system for lung cancer organoids culture (Figure 1). In their work, the ALI system could maintain the stroma cells like tumor-infiltrating immune cells to better recapitulate the TME (40). They firstly used this culture system for recapitulating the tumorigenesis in murine organoids models. Then they performed this system in lung cancer organoids (adenocarcinoma and squamous cell carcinoma) and other tumors for more than 100 tumor biopsies (40). They have successfully constructed 87% of lung cancer organoids for more than 100 days and modeled the immune checkpoint blockade (ICB). At this point, the growth of stroma cells may not be a barrier for organoids system establishment.

#### Lung Cancer Organoids Modeling for Understanding of Lung Cancer Biology

As a preclinical lung cancer system, organoids model maintains not only the genetic characteristics of parental lung cancer, but also retains the histology of original cancer tissues after long-term *in vitro* culturing. Tsao's study showed that after long-term expansion, lung cancer organoids models still can recapitulate the histology of the patient and the PDX models (35). The organoids xenograft models they constructed also maintain the tumorigenic character and the mutation of the original cancer tissues (35). Shi et al.'s research showed that even after more than 4 months 3D culturing *in vitro*, early stage(I/II) NSCLC organoids could still express the marker of their cancer type such as Ki76 and thyroid transcription factor-1, which supported the expansion of NSCLC organoids models use (5). The great recapitulation of original specimens can help us understand the mechanism and tumorigenesis of lung cancer.

#### Lung Cancer Organoids Models for Lung Cancer Clinical Application

##### Lung Cancer Organoids Models for Targeted Drug Evaluation and Personalized Medicine

Increasing studies have shown that organoids could be an excellent model for screening personalized cancer medicine. One such application is the use of organoids to evaluate patient-specific responses to cancer-fighting drugs. Organoid cultures maintain the epigenetic and genetic alterations of the original lung cancer, which could provide an excellent alternative to explore the drug resistance of the tumor that occur in patients. In one study, organoids with three different kinds of genetic mutations were developed to analyze the response to lung anticancer drug: organoids with BRCA2-mutant to Olaparib, organoids with EGFR-mutant to erlotinib, and organoids with EGFR-mutant/MET-amplified to crizotinib (36). The results showed that the IC50 of organoids with p.W2619C mutation in olaparib was lower than the organoids with p.M965I mutation. The structure of organoids with p.W2619C mutation was destroyed after olaparib treatment. The findings were consistent with the PDX model and also the hypothesis that p.W2619C is a pathogenic mutation that plays a significant role in synthetic lethal with poly (ADP-ribose) polymerase (PARP) inhibition (41). The organoids of EGFR-mutant with or without MET-amplified also showed patient-specific drug responses to erlotinib and crizotinib, and the PDX model showed similar results (36). In addition, the therapy efficacy is poor for patients with NSCLC with EGFR Exon 20 insertion mutation, and there is no approved targeted drug for this new mutation (42). Cho et al.'s team have established several preclinical models like PDXs, organoids, and patient-derived cells to examine the drug response of Amivantamab, which is a EGFR-MET bispecific antibody (43). Their results were consistent with the ongoing phase I study, indicating that amivantamab could be a promising targeted drug for EGFR Exon 20 insertion mutation patients (43).

**Figure 2:**
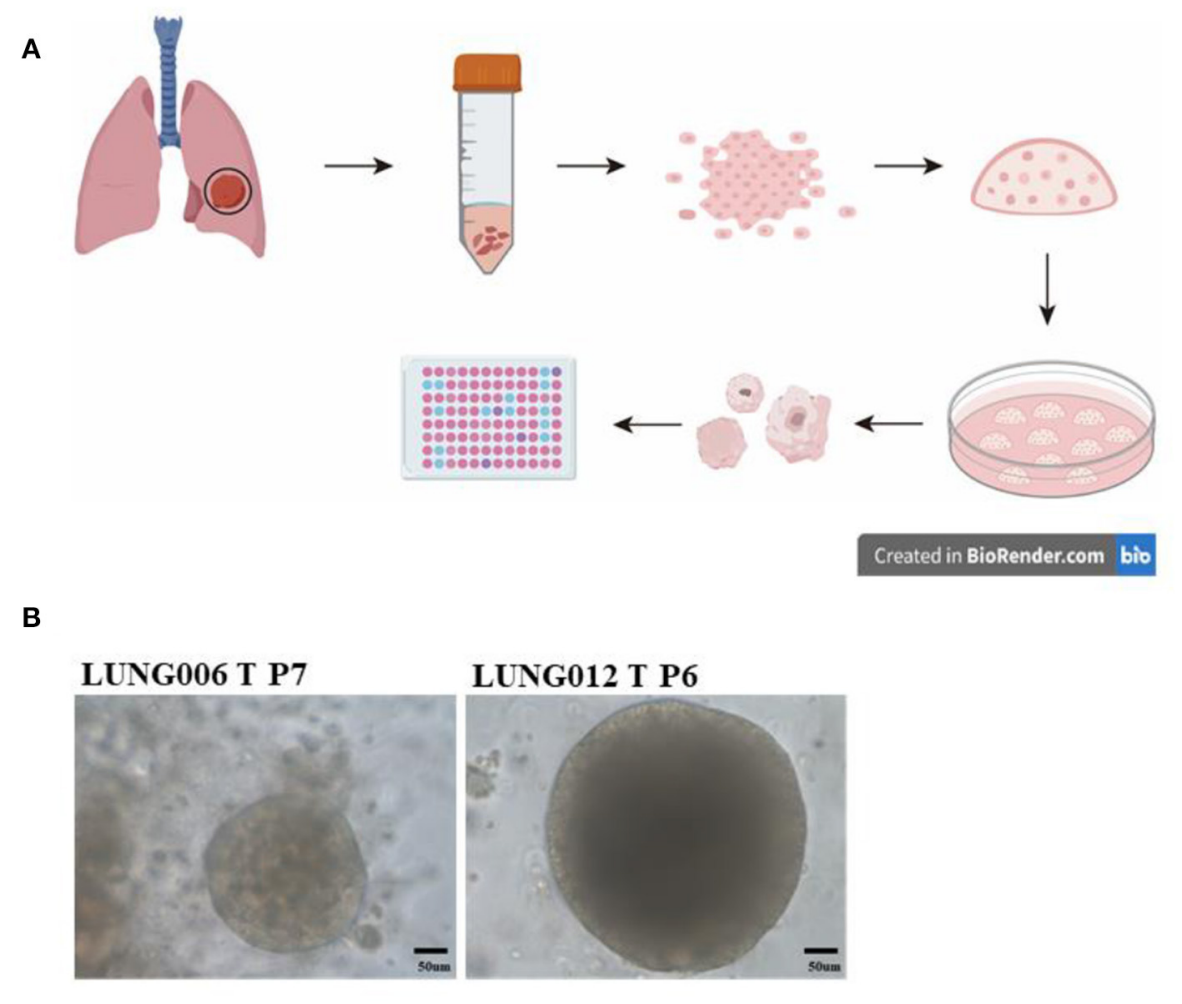
Drug testing *via* patient-derived organoids models: **(A)** Collecting the lung cancer specimens from patients during surgery, digesting the tissue and seeding cells in growth factors reduced Matrigel; dome culturing in culture medium; organoids culturing and passage; preforming Cell Titer-Glo Luminescent cell viability assay for drug testing. **(B)** Representative image of long-term culture lung cancer organoids (more than five passages) in our lab.

Organoids models could also be used to explore the effective target therapy for patients NSCLC with HER2 mutations. Previous studies showed that HER2 mutations occur in ~2–3% of NSCLC (44). Most of the NSCLC patients with HER2 mutation showed limited effect to HER2 inhibitors like afatinib, dacomitinib, neratinib, and trastuzumab (45–48). Hirsch et al.'s work showed that HER2 inhibitor pyrotinib is effective for patient-derived organoids established from HER2 exon 20 mutations patients, as well as the corresponding PDX models (49). After that, a phase II clinical trial showed that patients with HER2 mutation displayed promising drug sensitivity to pyrotinib (50). Another group established the *in vitro* patient-derived lung cancer organoids models and explored the drug response from different classes of molecular-targeted drugs, like small-molecule inhibitors, monoclonal antibodies, and an antibody–drug conjugate. In their experiments, HER2 inhibitors were also evaluated using organoids models, and their results indicated that patient-derived organoids is a suitable preclinical model for molecular targeted drug screening and evaluation (51). These studies indicated that although organoids models were cultured *in vitro*, the 3D culturing method could capture the genetic and tumor biology of original cancer, just like PDX models, but at lower cost and less time. It also provides a significant way for identifying promising therapies and drug screening (Figure 2).

##### Lung Cancer Organoids Models for Immunotherapy

In addition to targeted therapies, lung cancer organoids models also could be used to model the tumor-immune microenvironment. Since one of the weakness of traditional organoids model is the lack of TME, some studies have developed the new organoids culture technique, the coculture systems with additional cellular components such as tumor-infiltrating lymphocytes (TILs) (52). For example, previous studies showed that the key to simulate the immune checkpoint blockade is to preserve the original tumor T cell receptor spectrum (53). Voest et al.'s study showed that when peripheral blood lymphocytes coculture with autologous lung cancer organoids it could expand and enrich the tumor-reactive T cells (54). In their work, after processing specimen from patients with lung cancer, organoids models were cocultured with the patient' s peripheral blood lymphocytes. Some supplements like IFNγ, IL2, and anti-PD1 antibodies were added to promote the expansion and enrichment of tumor-reactive CD4 and CD8 T cells. Subsequently, organoids were used to evaluate the immune reactivity of these tumor-reactive T cells in various assays (54). Voest et al.'s study also showed a T cells coculture system in NSCLC, which could activate the tumor-reactive CD8 T cells (55). Furthermore, combination of anti-PD-L1 and MEK inhibitor in NSCLC organoids models could predict the drug response of clinical patients (56). These organoids studies indicated the significant role of organoids models in immunotherapy of lung cancer.

##### Lung Cancer Organoids Models for Drug Screening and Biomarker Screening

In the past decades, plenty of lung cancer drug studies performed in standard 2D lung cancer cell lines models were proven to be ineffective because of the genetic drift and clone selection in lung cancer cell lines (57). Organoids models could well retain the physiological architecture of original specimens, specific function of native tumor, and also the simulation of drug responses. These features allow organoids to be a better preclinical model for drug screening, exploration of novel molecular targeting, and study of potential mechanisms involved in the acquisition of drug resistance. For instance, a previous work has compared the drug sensitivity and drug resistance of lung cancer cell lines between culturing as a lung multicellular tumor spheroids (MTS) way and a monolayers way (58). Their result showed a very different drug response between 2D and 3D cultures. Conrad et al.'s study has performed genomic analysis to compare drug effect of patient-derived lung cancer cells between the monolayer models and matrix-dependent organoids models. Their results showed that the novel link between drug sensitive and DNA repair deficiency was found in organoids, but undetectable in monolayers during the drug-induced cell death and growth arrest (59). Besides, another work showed that patient-derived lung cancer organoids models were amenable to further -throughput drug screening because of its well-retaining ability of tumor histopathology and cancer gene mutation (39). So far there are multiple studies that have successfully constructed different subtypes of lung cancer organoids. For example, Jang et al.'s team has established five histological subtypes of lung cancer for more than 80 lung cancer organoids (36). The long-term culture organoids established by Tsao' team also confirmed the genomics and biological similarity between lung cancer organoids and the original tumor (35). These results indicated that lung cancer organoids system could be a potential promising platform for drug screening and biomarker validation (35, 36).

### Limitation of Lung Cancer Organoids Models

Although lung cancer organoids could be a promising drug screening tool, a potential biomarker bank, and a great model for drug evaluation, this advanced model has its limitations. The most important limitation is the culture methodology, which can hinder organoids models from different applications. Although the culturing medium could supply nutrition for organoids growth and development, there will be a potential possibility that the growth factor like small molecule inhibitors in culture medium may change the drug response of the organoids. Furthermore, it remains unclear to what extent this molecule will impact the tissue maturation of lung cancer organoids. Despite Matrigel being the most common extracellular matrix *in vitro* for organoids models, there is still abundant of growth factors that may affect the differentiation and growth of organoids like molecular in culture medium (60). In addition, the progression in growth of organoids can vary wildly, which is a major problem for high throughput drug screening (61). The lack of blood vessels in the microenvironment of organoids to remove waste and dead cells is another challenge for culturing of organoids, which may limit the growth size of organoids. Some studies have tried to incorporate endothelial cells or perform engineered culture technology to solve this problem (62). Moreover, research about parenchyma–stromal cocultures is rare, which may limit our understanding of their long-term effect in tumor growth and drug responses (63). Lastly, performing complex technology using organoids like CRISPR Cas9 technology or construction of lentivirus vector is not as convenient as lung cancer cell lines, which limits its applications in lung cancer research (64).

## Conclusion

Lung cancer cell lines are easy to handle, fast growing, and have a low cost for lung cancer research and large-scale studies. However, the lack of tumor heterogeneity, cell–cell interaction, and monolayer in culture have limited their translational application in medicine. *In vivo* lung cancer model PDX could retain better the TME. PDX models provide a solution for the lack of tumor heterogeneity and cell–cell interaction. Researchers could perform tumor size measurements and toxicity examination to directly observe the drug response. Nevertheless, the limited passage and high cost, and also the time consuming nature of the PDX model hamper large-scale studies and widespread use in a range of experiments. Lung cancer organoids could better maintain the biology and genetic characteristics of the original cancer, which allows researchers to perform drug evaluation and biomarker identification. Its 3D culturing method could well mimic the microenvironment of the initial tumor. In addition, organoid models could save a lot of time when compared with PDX models, which could more rapidly provide answers that benefit lung cancer patients in the clinical setting. It also makes co-culture with other cell types possible, giving researchers more opportunities to study the microenvironment and tumor immunology. Furthermore, researchers could also knock down/out or overexpress the potential gene in organoids for functional and lethality explorations.

While organoids models have many advantages, a major obstacle for lung cancer research would be the establishment of organoids models and their standardization. Unlike other cancer models, lung cancer organoids are more difficult to initiate and maintain in the long-term propagation. It is also noted that the standards of successful establishment of organoids models vary between different laboratories with no definitive standardized methodology for lung cancer organoids models, even for the ECM ingredients, culture medium, or whether dome culture method should be used or not. Introducing stroma, immune cells, biomechanical stimuli, and blood vessels into organoids culture could be a promising way in future to overcome the problem of the lack of microenvironment, thereby improving the organoids model.

## Author Contributions

CN: conception and design. JC, RL, WC, AlW, and AiW: development of methodology and acquisition of data. YL: analysis and interpretation of data and writing the manuscript. CN and NW: review the manuscript. All authors contributed to the article and approved the submitted version.

## Funding

This work was supported in part by the Hong Kong Research Grants Council Research Impact Fund (Ref. R4017-18), and in part by Hong Kong Research Grants Council (RGC), General Research Fund (GRF) (no: 14119019).

## Conflict of Interest

The authors declare that the research was conducted in the absence of any commercial or financial relationships that could be construed as a potential conflict of interest.

## Publisher's Note

All claims expressed in this article are solely those of the authors and do not necessarily represent those of their affiliated organizations, or those of the publisher, the editors and the reviewers. Any product that may be evaluated in this article, or claim that may be made by its manufacturer, is not guaranteed or endorsed by the publisher.
